# Immunolocalization of cation-chloride cotransporters in the developing and mature spinal cord of opossums, *Monodelphis domestica*

**DOI:** 10.3389/fnana.2013.00012

**Published:** 2013-05-15

**Authors:** Ha-Loan Phan, Jean-François Pflieger

**Affiliations:** Département de Sciences Biologiques, Université de MontréalMontréal, QC, Canada

**Keywords:** cation-chloride cotransporters, development, inhibition, locomotion, marsupial, motoneurons, spinal cord

## Abstract

Spinal inhibition is required to generate coordinated outputs between antagonistic muscles during locomotion. It relies on low neuronal chloride concentration set by two cation-chloride cotransporters, NKCC1 and KCC2 which, respectively, pumps Cl^−^ in or out of neurons. It is generally accepted that NKCC1 is gradually inactivated during development, while KCC2 is upregulated and activated, resulting in low intracellular [Cl^−^]. Newborn opossums are very immature but perform rhythmic and alternate movements of the forelimbs to crawl on the mother's belly and attach to a teat. Their hindlimbs are immobile. The alternation of the forelimbs suggests that mechanisms allowing spinal inhibition are present at birth. We studied the anatomical basis of inhibition in the spinal enlargements of postnatal opossums by immunolocalizing NKCC1 and KCC2. In some specimens, motoneurons and sensory afferents were labeled with TRDA prior to immunolabeling. At birth, both NKCC1 and KCC2 are detected in the presumptive gray and white matter of the ventral and the intermediolateral cord of both enlargements, but are sparse in the dorsal horn, where KCC2 is mostly seen on a small bundle of dendrites along primary afferents. KCC2 labeling is bright and has a mesh-like appearance in the gray matter and a radial appearance in the white matter, whereas NKCC1 is pale and diffuse. The subsequent expression of the cotransporters follows general ventrodorsal and mediolateral gradients, with the lumbar segments slightly lagging the cervical segments, until the mature pattern is observed around the 5th week. At all ages studied, KCC2 labeling is strong in the periphery of neurons. NKCC1 labeling decreases and becomes more uniformly distributed in the cells with age. Despite the significant anatomical and motor differences between the forelimbs and the hindlimbs of neonatal opossums, the maturation of KCC2 and NKCC1 is quite similar in both enlargements.

## Introduction

Alternation between limbs requires reciprocal inhibition of motoneurons and interneurons that constitute the central pattern generators located on both sides of the spinal cord (Grillner and Wallen, 1985; Vinay and Jean-Xavier, 2008; Nishimaru and Kakizaki, 2009). In the adult mammalian spinal cord, inhibition is driven by two major neurotransmitters, GABA and glycine. Their inhibitory effect depends on the intracellular concentration of chloride ions ([Cl^−^]_i_). In mature neurons, due to low [Cl^−^]_i_, the activation of GABA_*A*_- and glycine-receptor-gated chloride channels leads to an influx of Cl^−^ and to hyperpolarization of the membrane potential. In contrast, immature neurons have a high [Cl^−^]_i_ and instead respond to GABA and glycine stimulation by depolarizing their membrane (Delpire, 2000; Mercado et al., 2004). A lowering in neuronal [Cl^−^]_i_ occurs during development, causing a switch from depolarization to hyperpolarization (reviewed in Ben-Ari, 2001; Vinay and Jean-Xavier, 2008; Blaesse et al., 2009; Ben-Ari et al., 2012). At least two major cation-chloride cotransporters control [Cl^−^]_i_: the sodium-potassium-chloride cotransporter isoform 1 (NKCC1), which pumps extracellular Cl^−^ into neurons, and the potassium-chloride cotransporter isoform 2 (KCC2), which extrudes Cl^−^ from neurons. The lowering of [Cl^−^]_i_ which brings the switch from the immature to the mature response of neurons to GABA and glycine stimulation leads to believe that a developmental change could happen in the two cotransporters. The ontogenetic expression of NKCC1 and KCC2 in the spinal cord of perinatal rodents has been studied by immunohistochemistry, *in situ* hybridization and electrophysiology (Hübner et al., 2001a; Kanaka et al., 2001; Li et al., 2002; Delpy et al., 2008; Stil et al., 2009, 2011). These reports reveal that both cotransporters are expressed early in development but NKCC1 is predominant, keeping a high [Cl^−^]_i_ in neurons. Its activity becomes reduced later on while KCC2 becomes predominant, bringing on the switch from depolarizing to hyperpolarizing inhibitory response that occurs around birth in rodents.

Like all marsupials, opossums *Monodelphis domestica* are born more immature than rodents. After 14.5 days of gestation (Fadem et al., 1986), the newborn opossum must travel on the mother's belly from the urogenital opening to a teat where it remains attached for more than 3 weeks. Without help from the mother, the newborn uses its forelimbs to climb, grasping fur in alternate and rhythmic crawling movements while the trunk sways from side to side. By contrast, its hindlimbs are immobile; they start moving during the second week (Pflieger et al., 1996). The existence of alternate forelimb movements in newborn opossums, which has been quantified following electrical and pharmacological stimulation in *in vitro* preparations (Amalric et al., 2012), suggests that inhibition already exists in the cervical spinal cord, but this may not yet be the case in the lumbar cord in view of the hindlimbs immobility. We have investigated the ontogenic expression of NKCC1 and KCC2 in the spinal enlargements of newborn and postnatal opossums by immunohistochemistry. The present study is a step toward characterizing the emergence of spinal inhibition in this species and gaining a better understanding of the development of motor systems in marsupials, which face specific challenges due to their precocious birth. Preliminary results have been published in abstract form Phan and Pflieger (2009, 2010).

## Materials and methods

### Animal and tissue preparation

This research was performed under the guidelines of the Canadian Council on Animal Care using protocols approved by the University Ethics Committee.

A total of 48 opossums was used (5 at P0, day of birth, 5 at P5, 10 at P10, 5 at P12, 6 at P15, 1 at P25, 2 at P30, 2 at P40, 1 at P50, 1 at P60, 10 adults). Animals were obtained from a colony maintained at the University animal facility according to Fadem et al. (1986) (for details, see Cassidy et al., 1994; Pflieger et al., 1996).

Opossums younger than P25 were deeply anesthetized by hypothermia, beheaded and eviscerated in 0.1 M PBS (phosphate-buffered saline). A laminectomy was performed to expose the spinal cord before immersion in a fixative made of 4% paraformaldehyde in 0.1 M PBS for 10–24 h at 4°C. Animals aged P25 and older were administered an overdose of sodium pentobarbital by intraperitoneal injection and perfused transcardially with PBS followed by the above fixative. The cervical and lumbar enlargements of the spinal cord were left in the cartilaginous vertebrae of <P15 animals or dissected out in older animals, and the tissues were bathed in the fixative for 4 h at 4°C before being transferred to a 30% sucrose solution in 0.1 M phosphate buffer overnight at 4°C for cryoprotection. The tissues were embedded in Tissue-Tek OCT Compound (Miles Scientific) and solidified using powdered dry ice. Serial transverse sections (16 μm-thick) were cut on a cryostat (CM3050S Leica) and mounted on Superfrost slides (Fisher).

### Immunohistochemistry

**Figure 1:**
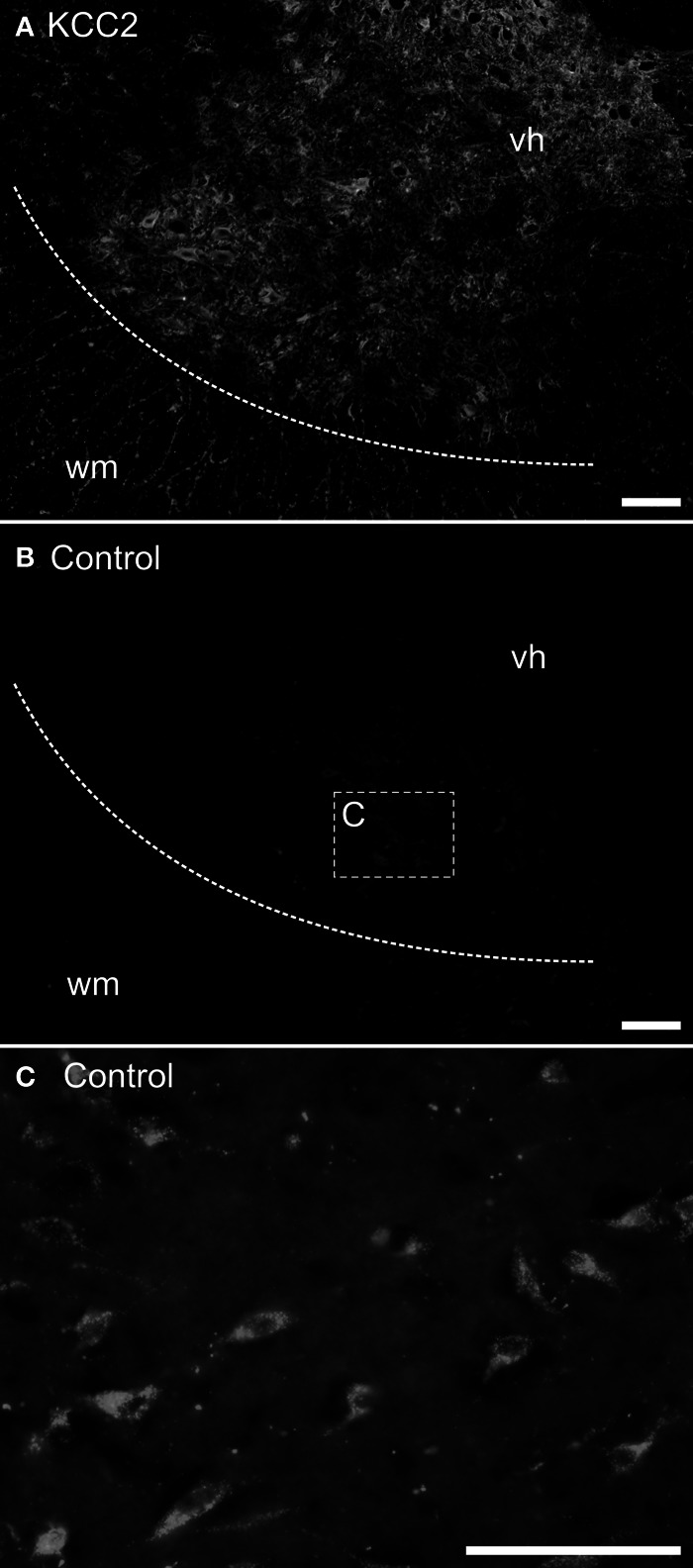
**Control experiments for immunohistochemistry.** Transverse sections of an adult opossum spinal cord processed with **(A)** or without **(B,C)** the KCC2 primary antibody. The boundary between the white and the gray matter is indicated by a dashed line and the box in **(B)** indicates the approximate location of the photomicrograph in (C). **(C)** Neurons showing auto-fluorescent granules in their cytoplasm. All sections were photographed at the same exposure time. Scale bars = 100 μm.

The mounted sections were washed 20 min at room temperature in 0.05 M Tris buffer with 1.5% NaCl and 0.3% Triton X-100 (Sigma), pH 7.4 (TBST) and incubated overnight at 4°C with either a purified rabbit polyclonal anti-KCC2 antibody (1:400) or a mouse T4 monoclonal anti-NKCC1 antibody (1:400); see below about antibodies. The sections were washed three times in TBST and incubated 2 h at room temperature in TBST with 3% BSA (Bovine Serum Albumin) and 10% NGS (Normal Goat Serum) containing either a goat antirabbit secondary antibody coupled to Alexa-Fluor488 (1:400; Invitrogen) or a goat antimouse secondary antibody coupled to Cy3 (1:400; Jackson Immunosearch). The sections were then washed once in TBST and twice in 0.05 M Tris buffer, and coverslipped using Fluoromount (SouthernBiotech) as a mounting medium. Controls were performed by omitting either the primary or the secondary antibody. No labeling was observed under these conditions (compare Figures 1A,B).

Neurons acquire endogenous fluorescence with age (Figure 1C) that can interfere with fluorescent labeling. Therefore, in addition to the immunofluorescence experiments described above, the slides from some adult specimens were processed for immunohistochemistry using diaminobenzidine (DAB) to reveal the presence of the chloride cotransporters. These sections were washed three times in TBST for 30 min each and, to reduce non-specific labeling, they were blocked with 1% NGS, 3% BSA, and 0.2% Triton X-100 (Sigma) in 0.1 M PBS for 30 min before incubation with the primary antibody (either anti-KCC2 or anti-NKCC1). The sections were rinsed and incubated with a biotinylated horse-antirabbit antimouse IgG (1:200; Vector kit) for 1 h at room temperature. After rinsing, an avidin-biotin-peroxidase complex solution (1:200; Vectastain ABC kit, Vector laboratories) was applied for 1 h at room temperature, and revealed using DAB peroxidase precipitation for 2–5 min. The marker appears as dark dots visible in bright field microscopy. After final washes to halt peroxidase precipitation, the sections were air-dried overnight, dehydrated with increasing concentration of ethanol and then toluene, and finally coverslipped with Eukitt (Electron Microscopy Science) as mounting medium. Controls were performed as described above.

The KCC2 antibody used here was directed against rat KCC2/Slc12a5 (Upstate Millipore, product number 07–432) and has been used for similar studies in rodents (Delpy et al., 2008; Stil et al., 2009, 2011; Horn et al., 2010). Protein sequences of rat KCC2/Slc12a5 (NP_599190) and its opossum ortholog (XP_001366304) share 96% similarity. The 112 amino acid epitope targeted by our antibody is 86% similar in the two species. The NKCC1 T4 monoclonal antibody used here was directed against human NKCC1/Slc12a2 (Developmental Studies Hybridoma Bank, University of Iowa, USA), and has been successfully employed in prior studies conducted in mice (Delpy et al., 2008). This antibody also recognizes the NKCC2/Slc12a1 isoform (Lytle et al., 1995) but the latter is not expressed in the spinal cord (see Stil et al., 2009), suggesting that our antibody specifically labels NKCC1 in the opossum's spinal cord. Protein sequences of human NKCC1/Slc12a2 (AAI46840) and its opossum ortholog (XP_003341631) share 88% similarity. The 225 amino acid epitope targeted by our antibody is 85% similar in the two species.

### Texas red tracing

In order to label motoneurons innervating the limbs, tracing with Texas Red coupled to dextran amine (TRDA, 3000MW, Invitrogen) was performed in 2 newborn and 2 P12 opossums using *in vitro* preparations as developed by Lavallée and Pflieger (2009). This also labeled primary afferents growing into the cord. Dissections were performed in a dish filled with a physiological solution (NaCl 125 mM, KCl 3 mM, NaHCO_3_ 25 mM, NaH_2_PO_4_ 1 mM, MgCl_2_ 1 mM, CaCl_2_ 2 mM, dextrose 15 mM, oxygenated with 95% O_2_/5% CO_2_; pH 7.4). After anesthesia and evisceration, the neuraxis was transected at the obex. The dorsal skin and muscles were then removed and a laminectomy was performed. Nerves of the brachial and lumbar plexi on one or both sides were exposed. The preparations were removed from the physiological solution to allow drying of the nerves while keeping the spinal cord wet. TRDA crystals were applied to the freshly cut nerve stumps for 3–4 min to allow intake of crystals by the cut fibers. The specimens were then bathed in renewed oxygenated physiological solution in the dark for 4–8 h, depending on the age. Afterwards, the tissues were transferred to the above fixative solution and processed for KCC2 or NKCC1 immunohistochemistry.

### Fluorescence microscopy

The sections were observed with an Olympus microscope BX51 equipped for fluorescence with an FITC filter (for Alexa-488, excitation 450–490 nm, emission 520 nm) and a Rhodamine filter (for Cy3 and TRDA, excitation 510–560 nm, emission 590 nm). Photographs were acquired using a digital color camera (QImaging) controlled by an imaging software (Image-Pro Plus, Media Cybernetics). When needed, contrast, gamma, and brightness of the photomicrographs were manually adjusted with Image-Pro Plus. Photographs used for fluorescence intensity analysis (see below) were left untouched. The figures were edited in Corel Draw (Corel).

In the TRDA tracing experiments, photomicrographs of the ventral cord where motoneurons are located were successively taken with a Rhodamine filter (TRDA labeling in magenta) and a FITC filter (cotransporters labeling in green) in order to determine the cellular distribution of the cotransporters (Figure 6A1). The TRDA marker fills the motoneuronal cell bodies, reaching its maximal intensity at the center of the cell and decreasing at the periphery, and helps appreciate the cell morphology at ages when motoneurons are undifferentiated. Using the *Intensity analysis function* of the imaging software, the respective fluorescence intensities for TRDA and the cotransporters were measured along a line placed on each motoneuron sufficiently labeled for measurement (on various sections of the brachial cord; Figure 6A2). Fluorescence data were exported to Prism (GraphPad). To allow comparison between different motoneurons, the curves for both TRDA and one or the other cotransporter were normalized in fluorescence/*y*-axis (where 0% corresponds to an absence of fluorescent intensity and 100% corresponds to the maximal intensity measured for a given motoneuron), superimposed, and then normalized in length/*x*-axis (where 0 and 100% of the cell length correspond, respectively, to 50% of fluorescence on the ascending and the descending parts of the TRDA curve; see Figure 6A3).

## Results

### KCC2 and NKCC1 localization in the developing and mature spinal cord

**Figure 2:**
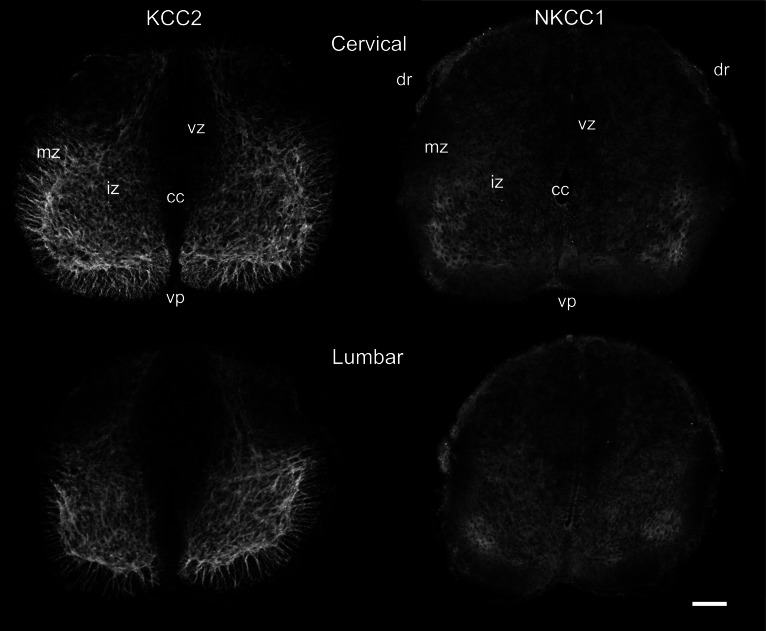
**Distribution of KCC2 and NKCC1 in the P0 spinal cord.** Transverse sections of the cervical **(upper row)** and the lumbar **(lower row)** enlargement, processed to reveal KCC2 **(left column)** and NKCC1 **(right column)** localization. Scale bar = 50 μm.

**Figure 3:**
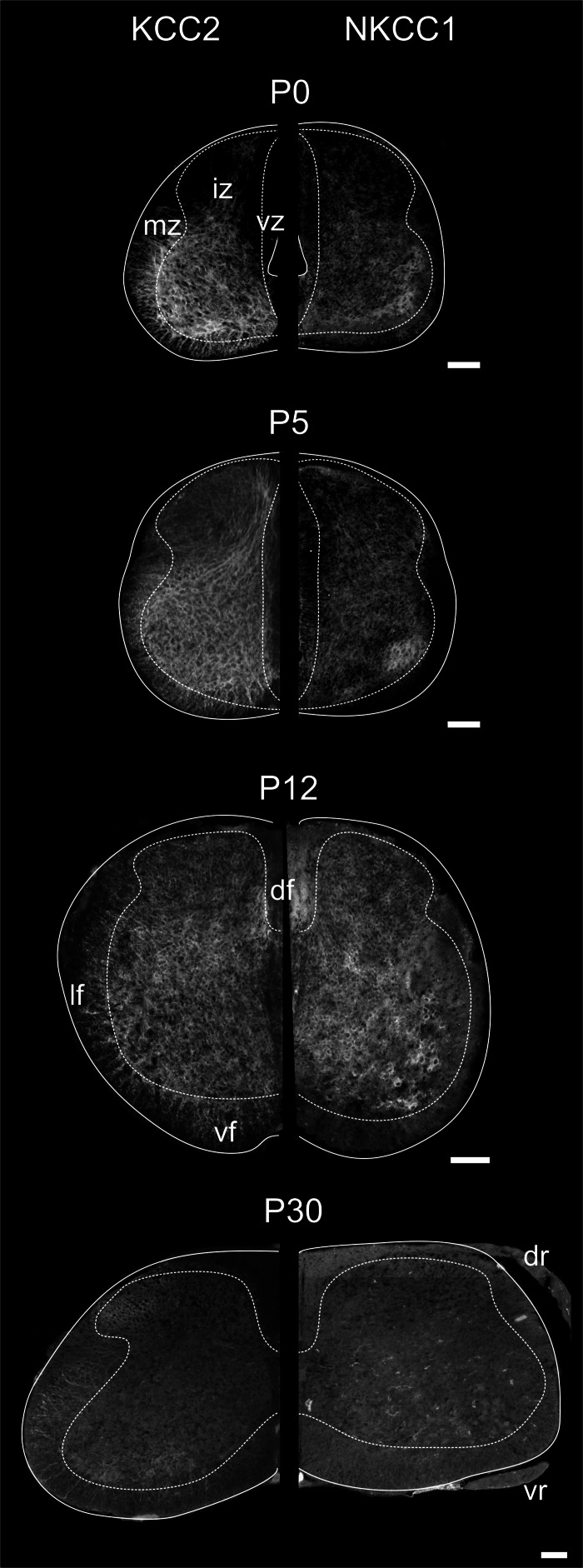
**Distribution of KCC2 and NKCC1 in the developing spinal cord.** Transverse hemisections of the cervical cord at selected ages, processed to reveal KCC2 **(left half)** or NKCC1 **(right half)**. Dashed lines delineate approximately the boundary between the white and the gray matter. Scale bar = 100 μm.

In newborn opossums, the spinal cord has an elongated central canal surrounded by a thick ventricular zone (vz in Figures 2, 3), suggesting that cell production and migration are still in progress. The intermediate zone, or presumptive gray matter (iz in Figures 2, 3), contains small and undifferentiated cells, many of them oriented radially, and no distinct neuropile. The marginal zone, or presumptive white matter (mz in Figures 2, 3), is very thin, especially dorsally. These immature features are more pronounced in the lumbar enlargement than in the cervical enlargement. Nonetheless, KCC2 and NKCC1 immunoreactivity is detected in both enlargements of the cord in newborn opossums (Figure 2).

In the spinal enlargements of newborn opossums, KCC2 immunoreactivity is present in the intermediate and marginal zones, but not in the ventricular zone, although the demarcation between the ventricular zone and the intermediate zone is not sharp (Figure 3). In the intermediate zone, KCC2 labeling is found in all of the ventral horn, where it is stronger at the margin where motoneurons are located, as well as in the medial and dorsal part of the dorsal horn where a small bundle of fibers could be distinguished. It is otherwise reduced or absent in the lateral part of the dorsal horn. The intermediate zone labeling is bright and has a mesh-like appearance. In the marginal zone, KCC2 labeling can be observed in the presumptive ventral and lateral funiculi, where it has a radial appearance, and in the dorsolateral and dorsal funiculi, where it is faint. The dorsal funiculi are barely formed in the newborn opossum (see Qin et al., 1993; Desrosiers, 1994). KCC2 labeling is very similar in both the cervical and the lumbar enlargements, but is slightly less pronounced caudally, most notably in the marginal zone.

Even though NKCC1 immunoreactivity is likewise distributed in both spinal enlargements of newborn opossums, it is strikingly less crisp and more diffuse than KCC2 immunoreactivity. It does not exhibit the mesh-like pattern in the intermediate zone or the radial pattern in the marginal zone observed for KCC2. Unlike KCC2 labeling clearly present along the entire margin of the ventral horn, NKKC1 labeling is only somewhat more intense in the ventrolateral portion of the ventral horn. NKCC1 labeling is less seen in the marginal zone, notably in the presumptive lateral funiculus, which is especially the case in the lumbar enlargement. However, it is found in the ventricular zone and the ventral plate as well as in the dorsal roots, all areas where KCC2 is absent.

Figure 3 illustrates the labeling for KCC2 (left) and NKCC1 (right) in the cervical cord of postnatal opossums. When comparing to P0, KCC2, and NKCC1 labeling at P5 extends to the dorsal and lateral regions of the dorsal horn. The intermediate zone has expanded at the expense of the ventricular zone, and the labeling for KCC2 extends more medially. Although less intense than in the ventral gray, the entire dorsal gray now shows at least diffuse labeling for KCC2. Immunolabeling continues ventrolaterally and delineates the dorsal horn from the intermediate gray matter. The density of NKCC1 immunoreactivity has slightly increased at P5. It remains diffuse and homogeneous, except for the denser spot in the ventrolateral ventral horn already observed at P0. NKCC1 is sparse in the presumptive white matter.

By P12, both KCC2 and NKCC1 are expressed homogeneously in the whole spinal gray. The ventricular zone is nearly reduced to an ependymal epithelium. The small bundle of KCC2 labeling in the medial dorsal horn described earlier becomes difficult to detect at P12. Radial KCC2-labeling is visible in the ventral and lateral funiculi, whereas dorsally the labeling is diffuse and punctate. NKCC1 labeling is now clearly seen in the presumptive white matter, except dorsolaterally (Figure 3).

**Figure 4:**
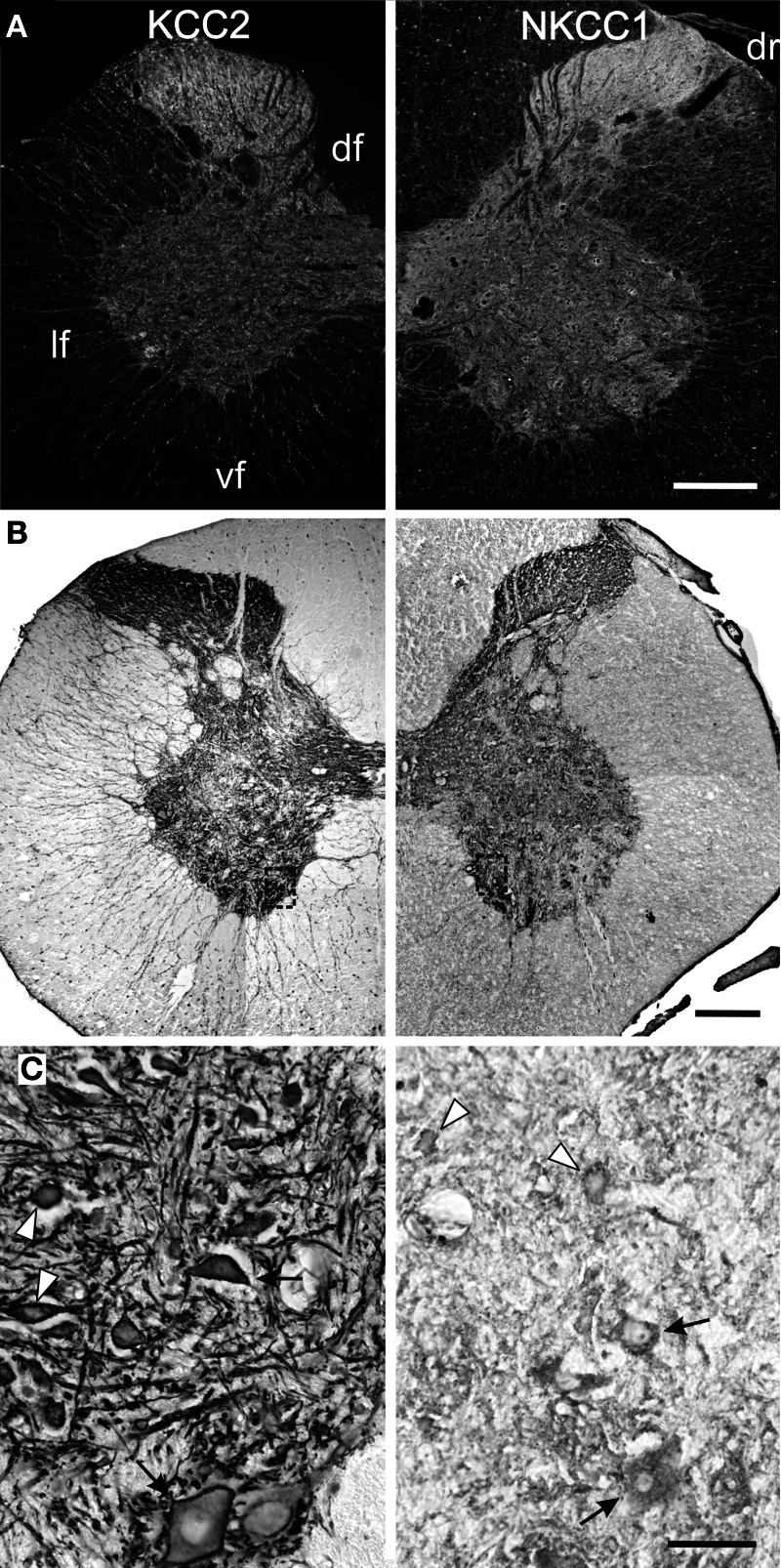
**Mature patterns of KCC2 and NKCC1 distribution.** Transverse hemisections of the cervical cord in the adult opossum processed to reveal KCC2 (left half) or NKCC1 (right half), using fluorescent antibodies **(A)** or DAB **(B,C)**. The boxes in **(B)** indicate the approximate location of the photomicrographs in **(C)**. **(C)** Motoneurons (arrows) and interneurons (arrowheads) in the medial ventral horn. Scale bars: in **(A,B)** = 2 mm; in **(C)** = 200 μm.

The labeling continues to spead at P15 and P25 and, from P30 onwards, KCC2 and NKCC1 immunoreactivities are widely distributed in the gray matter and the white matter (Figures 3, 4A), being more intense in the superficial dorsal horn than elsewhere in the gray matter, probably because of the high density of cell somata. This is particularly clear in the adult spinal cord, on sections processed either with immunofluorescence or DAB (Figure 4B). Mature labeling patterns for both cotransporters are reached by P40. At this stage, both motoneurons (arrows in Figure 4C) and interneurons (arrowheads in Figure 4C) show stronger labeling for KCC2 than for NKCC1. KCC2 labeling is also stronger in the periphery of neurons, often delineating the contour, while NKCC1 labeling is evenly distributed throughout the cytoplasm.

### KCC2 and NKCC1 in relation to motoneurons and primary afferents

Even though both cotransporters become distributed in all areas of the gray matter during development, their presence is observed first in the ventral horn, notably in the area of motoneurons. This is particularly true for KCC2, which labeling is stronger all along the margin of the ventral horn and which radial labeling in the presumptive white matter reminds of motoneuronal dendrites. In order to better localize KCC2 and NKCC1 in relation to motoneurons, the motoneurons of the lateral motor column were identified in some newborn specimens, when the cotransporters are mostly in the ventral horn, and at P12, when the cotransporters are more distributed in the gray matter. This was done by TRDA application to the cut nerves prior to immunohistochemical processing for the cotransporters.

**Figure 5:**
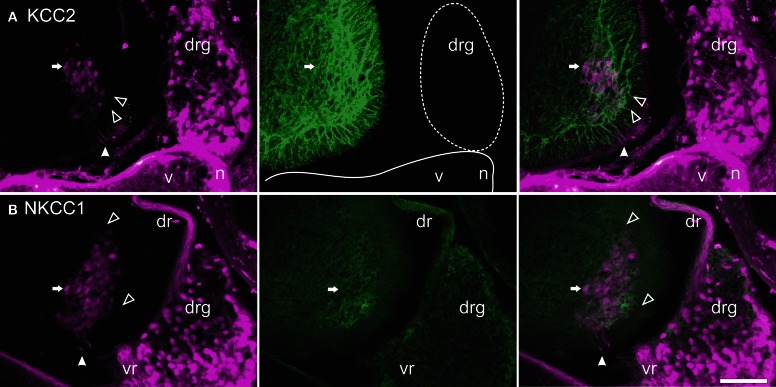
**Retrograde labeling with TRDA at P0.** Transverse sections of the cervical cord and adjacent tissues after application of TRDA (in magenta) to the brachial nerves prior to KCC2 (**A**, in green) or NKCC1 (**B**, in green) immunolabeling. Photomicrographs were taken with an FITC (left panels) or a Rhodamine filter (center panels) and were merged (right panels). A reference motoneuron is indicated by an arrow. Solid arrowheads indicate the ventral root exit point; empty arrowheads point to motoneuron dendrites in the marginal zone. Scale bar = 50 μm.

**Figure 6:**
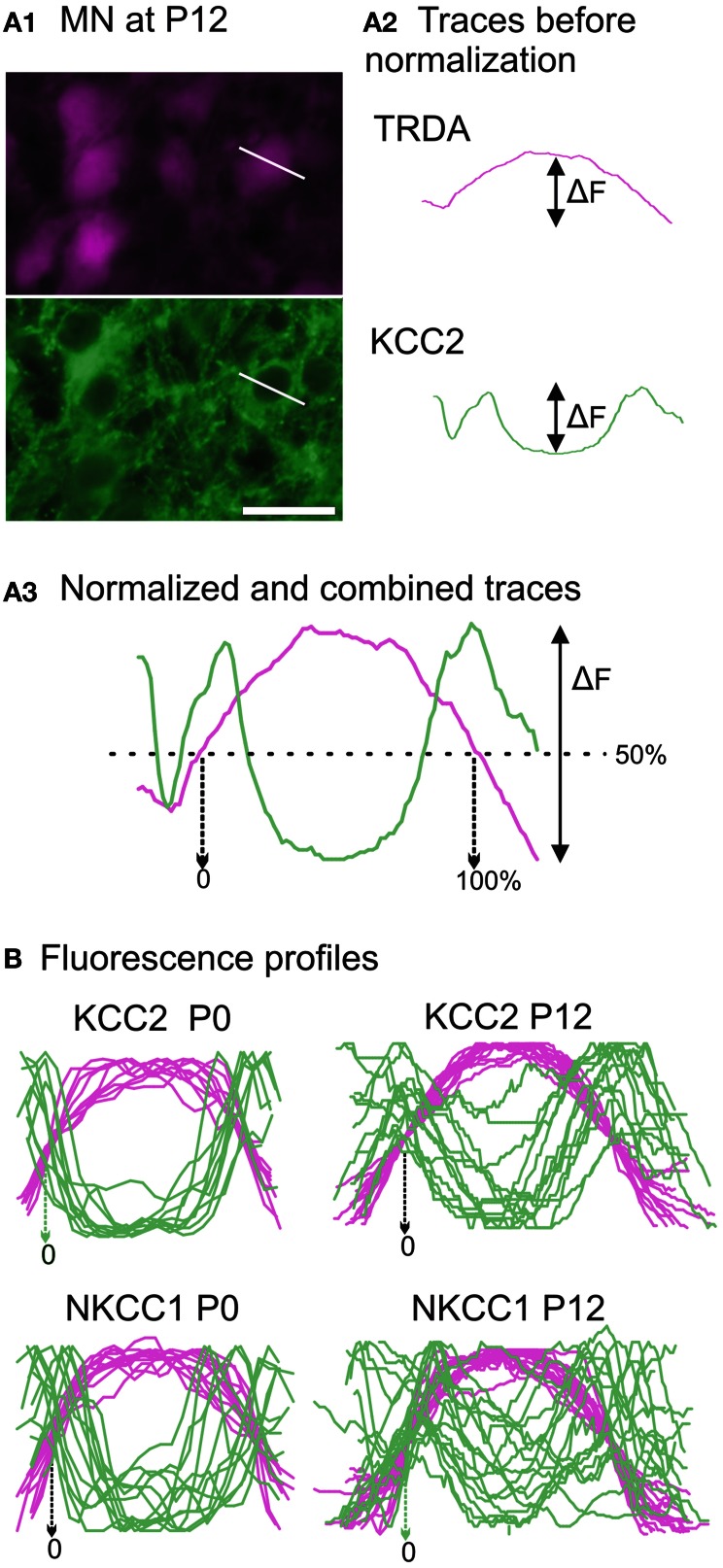
**Developmental distribution of KCC2 and NKCC1 in identified motoneurons. (A)** Photomicrographs of motoneurons taken with a Rhodamine filter for TRDA (in magenta) and with a FITC filter for KCC2 immunolabeling (in green) **(A1)**. The fluorescence intensities are measured along a line placed over a selected motoneuron. After curve reconstruction **(A2)**, TRDA intensity (in orange) and immunolabeling intensity (in green) curves are normalized along both axes and combined to produce a profile **(A3)**. **(B)** Superimposition of fluorescence profiles obtained at P0 (*n* = 10, left column) and P12 (*n* = 16, right column) for either KCC2 (upper row) or NKCC1 (lower row) immunolabeled neurons. Scale bar in **(A1)** = 25 μm.

At both ages, TRDA clearly labeled the somata and dendrites of motoneurons of the lateral motor column as well as their axons in the ventral spinal cord and the ventral roots (Figure 5). At P0, the exact location of the cotransporters within the TRDA-labeled motoneurons cannot be ascertained by looking at the slides under the microscope. However, when quantified with the Intensity analysis function of the imaging software, it is found that fluorescence intensity for both KCC2 and NKCC1 (Figure 6B, green curves) is minimal at the peak of the TRDA curves. Conversely, it is maximal at the ascending and the descending portions of TRDA curves (Figure 6B, magenta curves) in all motoneurons studied. At P12, the intensity of KCC2 labeling still peaks at the periphery in a majority of motoneurons. In contrast, the location of NKCC1 fluorescence varies more between motoneurons, suggesting a wider distribution within the cytoplasm.

**Figure 7:**
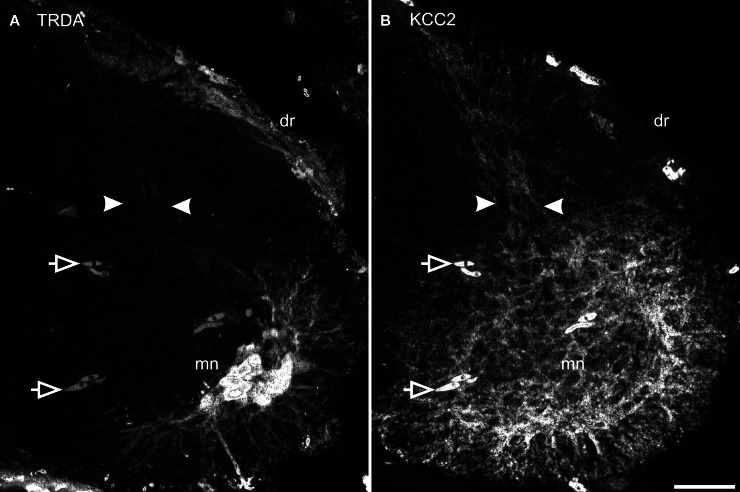
**Primary afferents superimpose with KCC2 immunolabeled neurites in the dorsal cord.** After TRDA application at the brachial plexus and KCC2 immunolabeling on a P0 cervical cord, TRDA labels motoneurons and primary afferents form the dorsal root running dorso-ventrally in the middle of the dorsal cord (**A**, between arrowheads). KCC2 is strongly expressed in the ventral spinal cord and by a small fascicle of neurites in the middle of the dorsal spinal cord (**B**, between arrowheads). Note that KCC2-ir is absent from the dorsal root. Arrows indicate erythrocytes. Scale bar = 30 μm.

TRDA also marked cell bodies in the dorsal root ganglia and primary afferents entering the cord by the dorsal roots. On transverse sections of the spinal cord of P0 opossums, primary afferents appear as a small fascicle that can be followed dorsoventrally into the dorsal horn, lateral to the ventricular zone (between arrows in Figure 7A), and into the ventral horn in close proximity to the dendrites of TRDA-labeled motoneurons (Figure 7A). These TRDA-labeled primary afferents are found in the same region as the small fascicle labeled for KCC2 described above (between arrows in Figure 7B). This observation was made until P12, after which the KCC2 fascicle could not be distinguished from background. The KCC2-labeled fascicle is very likely not made of primary afferents because ganglion cells and dorsal roots do not express KCC2 (see Figure 5A). They are probably dendrites from ventral neurons, but their origin could not be precisely identified due to the strong background of KCC2 labeling in the ventral cord.

## Discussion

This study describes the development of two major cation-chloride cotransporters in the spinal cord enlargements of the opossum, a mammal born very immature. The forelimbs of the newborn move in alternation and thus necessitate some reciprocal inhibitory spinal activity, but the hindlimbs are immobile until the middle of the second week. Therefore, it was expected that the respective pattern of expression of the cation-chloride cotransporters NKCC1 and KCC2 would be more mature in the cervical enlargement of the newborn than in the lumbar enlargement. It was also expected that in the following postnatal days or weeks, the lumbar segments would lag behind the cervical cord in its cotransporters labeling, as has been reported for other developmental events. Indeed the lumbar enlargement lags the cervical enlargement by 3–7 days for motoneuron development and cholinergic expression (Barthélemy and Cabana, 2005), synaptogenesis (Gingras and Cabana, 1999), myelinogenesis (Leblond and Cabana, 1997; Lamoureux et al., 2005), expression of the gap junction protein Connexin-36 (Lemieux et al., 2010), and manifestation of spontaneous activity (Lavallée and Pflieger, 2009). In contrast, we have found that, in the newborn opossum, the distribution of both cotransporters in the brachial cord is far from what it will become in the mature animal, and that each cotransporter is present in both enlargements in quite comparable respective pattern of distribution. Thus, considering the forelimb capability for alternate movements and the immobility of the hindlimbs of the newborn opossum, the spinal expression of KCC2 and NKCC1 is not well correlated with motor behaviors.

The general ventrodorsal and mediolateral gradients displayed in the developmental expression of the cotransporters, which are clearer for KCC2, match the general gradients of spinal neuron production and differentiation (Fujita, 1964; Altman and Bayer, 1984). Li et al. (2002) consider KCC2 expression to be an indicator of neuronal maturation. However, not all developmental events obey the inside-out gradient in the opossum cord. The opposite, an outside-in gradient, was evidenced in the expression of synaptic (Gingras and Cabana, 1999) and myelin (Lamoureux et al., 2005) proteins. The cotransporters labeling increases gradually in the superficial dorsal horn and remains strong as the mature pattern is acquired around P40. This contrasts with the gap junction protein Connexin-36, which shows a strong but transient expression in the dorsal horn between P10 and P20, and is nearly absent in the mature spinal cord (Lemieux et al., 2010). More importantly, immunohistochemical studies in developing opossums suggest that the spinal expression of glycine increases whereas GABA decreases along a ventrodorsal gradient (unpublished observation).

The nervous system of the newborn opossum can be compared approximately to that of an E13.5 rat or an E11.5 mouse (discussed in Smith, 2006; Lavallée and Pflieger, 2009). With the same antibodies used here, NKCC1 has been detected as early as E11.5 in the lumbar spinal cord of mice (Delpy et al., 2008) and KCC2 at E11.5–12.5 in rats (Li et al., 2002) and mice (Hübner et al., 2001b; Li et al., 2002; Stein et al., 2004; Delpy et al., 2008). The cervical cord was not examined in these studies, but considering that neurons are produced 1–2 days earlier in the cervical cord than in the lumbar cord (mouse: Nornes and Carry, 1978; rat: Altman and Bayer, 1984), the same may hold true for the expression of the cotransporters. It is at E13.5 that the distribution of the cotransporters in the lumbar cord of rodents compares to that in the cervical cord of the newborn opossum. Overall, the early development KCC2 and NKCC1 seems similar in opossums and rodents. Stil et al. (2009) reported developmental changes in the expression of these cotransporters in the rat lumbar cord up to P20, whereas was saw them until P40 in the opossum. Thus, possibly, the development of chloride cotransporters is more protracted in the opossum.

Recordings of lumbar ventral roots in *in vitro* preparations of rats and mice during chemically-induced fictive locomotion revealed that ventral root bursts between the two sides of the cord become well alternate around E18.5 (Branchereau et al., 2000; Nishimaru and Kudo, 2000). The same probably happens about 2 days earlier in the cervical cord that is, at the age when these rodents compare to P3–5 opossums and when, in the latter, alternation of the forelimbs is already manifest. It is safe to assume that forelimb alternation started before birth in opossums, thus earlier than in rodents. KCC2 is the most involved isoform in the development of inhibition in the spinal cord (see Blaesse et al., 2009; Ben-Ari et al., 2012), but it has been shown recently, in developing mice that KCC3 plays a role in the hyperpolarization of Cl^−^ equilibrium potential in a subset of dorsal root ganglion cells (Lucas et al., 2012). Mechanisms other than the chloride cotransporters, such as extracellular K^+^ or HCO^−^_3_ concentration or compartmentalization of inhibitory synapses (see Jean-Xavier et al., 2007; Vinay and Jean-Xavier, 2008), may likewise influence the switch of spinal neuron response to inhibitory neurotransmitters.

Finally, our study revealed that, in young opossums, primary sensory afferents labeled with TRDA and a small bundle of spinal dendrites expressing KCC2 are both aligned dorsoventrally and restricted to the medial part of the dorsal horn. The origin of these dendrites could not be precisely identified in our material, except that they come from neurons in the ventral horn. However, they are probably not from lateral column motoneurons since their dendrites arborize ventrally and laterally (Figure 7A; see also Knott et al., 1999). Our material does not allow us to ascertain if there is contact between these TRDA-primary afferents and the KCC2-spinal dendrites. Nonetheless, this relationship at precocious ages may reflect growth interactions between these structures during the establishment of sensorimotor networks (see Knott et al., 1999; Kitchener et al., 2006), the KCC2-labeled dendrites could serve as a substrate to guide primary afferents to their ventral targets or, alternatively, the primary afferents could support growth of dendrites from ventral neurons. Is KCC2 directly involved in those events or is its presence on the dendrites neighboring primary afferents only coincidental? KCC2 has been shown to promote dendritic spine development (Li et al., 2007; Horn et al., 2010), but, to our knowledge, it has not been involved in dendritic growth or guidance so far. Identifying the cells from which the KCC2-expressing dendrites originate would be a step to address these questions.

### Conflict of interest statement

The authors declare that the research was conducted in the absence of any commercial or financial relationships that could be construed as a potential conflict of interest.

## Abbreviations

df: dorsal funiculus
dr: dorsal root
drg: dorsal root ganglion
gm: gray matter
iz: intermediate zone
KCC2: potassium-chloride cotransporter isoform 2
mn: motoneurons
mz: marginal zone
NKCC1: sodium-potassium-chloride cotransporter isoform 1
TRDA: Texas Red coupled Dextran Amine
vr: ventral root
vz: ventricular zone
wm: white matter.
